# Outpatient Emergency Department Use Among Publicly Insured Patients

**DOI:** 10.1001/jamahealthforum.2025.5916

**Published:** 2025-12-26

**Authors:** Maya Spencer, Christopher Toretsky, Renee Y. Hsia

**Affiliations:** 1 Department of Emergency Medicine, University of California, San Francisco; 2 Philip R. Lee Institute for Health Policy Studies, University of California, San Francisco

## Abstract

This cross-sectional study examines emergency department visits from 2008 to 2023 in California.

## Introduction

The emergency department (ED) serves as a first and last resort of care, especially for patients without insurance or outpatient access, such as Medicaid beneficiaries.^1,2,3^ In contrast, privately insured and Medicare populations are assumed to rely less on the ED given greater access to primary and specialist care.^1,2,3,4^ Since the Affordable Care Act, coverage expanded substantially through Medicaid and marketplace plans, yet ED visits (especially outpatient encounters) continued to rise.^2,3^ Despite literature on overall ED trends, to our knowledge few studies have differentiated outpatient from inpatient ED use across payer groups. This distinction is critical to understanding whether shifts in utilization reflect broader changes in care delivery, access constraints, or coverage-related patterns. We examined ED visits from 2008 to 2023 in California, focusing on discharged vs admitted visits across Medicare, Medicaid, private insurance, uninsured, and other populations.

## Methods

We studied all ED visits from 2008 to 2023 from licensed, general acute care hospitals in California using data from the California Department of Healthcare Access and Information, the State Health Access Data Assistance Center, and the US Census. This study adhered to the Strengthening the Reporting of Observational Studies in Epidemiology (STROBE) reporting guidelines. Use of deidentified data did not constitute human participants research per the institutional review board of the University of California, San Francisco; thus, institutional review board approval and informed consent were waived. ED visits were categorized as discharged (treat and release), also known as outpatients or admitted patients. Payers were grouped as Medicare, Medicaid, private, uninsured, or other/unknown. A linear regression tested for trends. Data were analyzed using R, version 4.1 (R Foundation); a 2-tailed *P* < .05 was considered statistically significant.

## Results

From 2008 to 2023, annual ED visits increased by 38% from 10.8 million to 14.8 million visits. Adjusted for population growth, visits increased from 294 to 380 visits per 1000 enrollees, representing a 29% rise. The Table shows that Medicaid visits increased the most by 33.3%, more than double that of Medicare’s 12.5% (vs 5.2% privately insured and 2.2% uninsured). As shown in the Figure, outpatient visits increased sharply while inpatient visits remained relatively flat.

**Table.** Emergency Department (ED) Visits

Payer	No.		2008 Visit rate (per 1000 enrollees)	2023 Visit rate (per 1000 enrollees)	Change in rate (2008-2023), %	*P* value
	2008 ED visits	2023 ED visits				
**Total**						
Medicare	2 136 449	3 476 435	484.29	544.70	12.47	.58
Medicaid	2 652 658	6 265 967	580.61	773.84	33.28	<.001^a^
Private	3 671 417	3 980 339	174.11	183.17	5.20	.08
Uninsured	1 697 534	678 824	271.19	277.04	2.16	.72
Other/unknown	620 458	419 090	54.09	35.19	−34.94	<.001^a^
**Outpatient**						
Medicare	1 334 146	2 463 975	302.43	386.07	27.66	.046^a^
Medicaid	2 317 400	5 652 188	507.23	698.03	37.62	.001^a^
Private	3 250 214	3 618 008	154.14	166.50	8.02	.17
Uninsured	1 549 280	646 900	247.50	264.01	6.67	.40
Other/unknown	575 547	363 045	501.78	304.82	−39.25	<.001^a^
**Admitted**						
Medicare	802 303	1 012 460	181.87	158.64	−12.77	<.001^a^
Medicaid	335 258	613 779	73.38	75.80	3.30	.04
Private	421 203	362 331	19.97	16.67	−16.52	<.001^a^
Uninsured	148 254	31 924	23.68	13.03	−44.97	<.001^a^
Other/unknown	44 911	56 045	39.16	47.06	20.17	.31

^a^ *P* < .05 indicates statistical significance.

**Figure.**
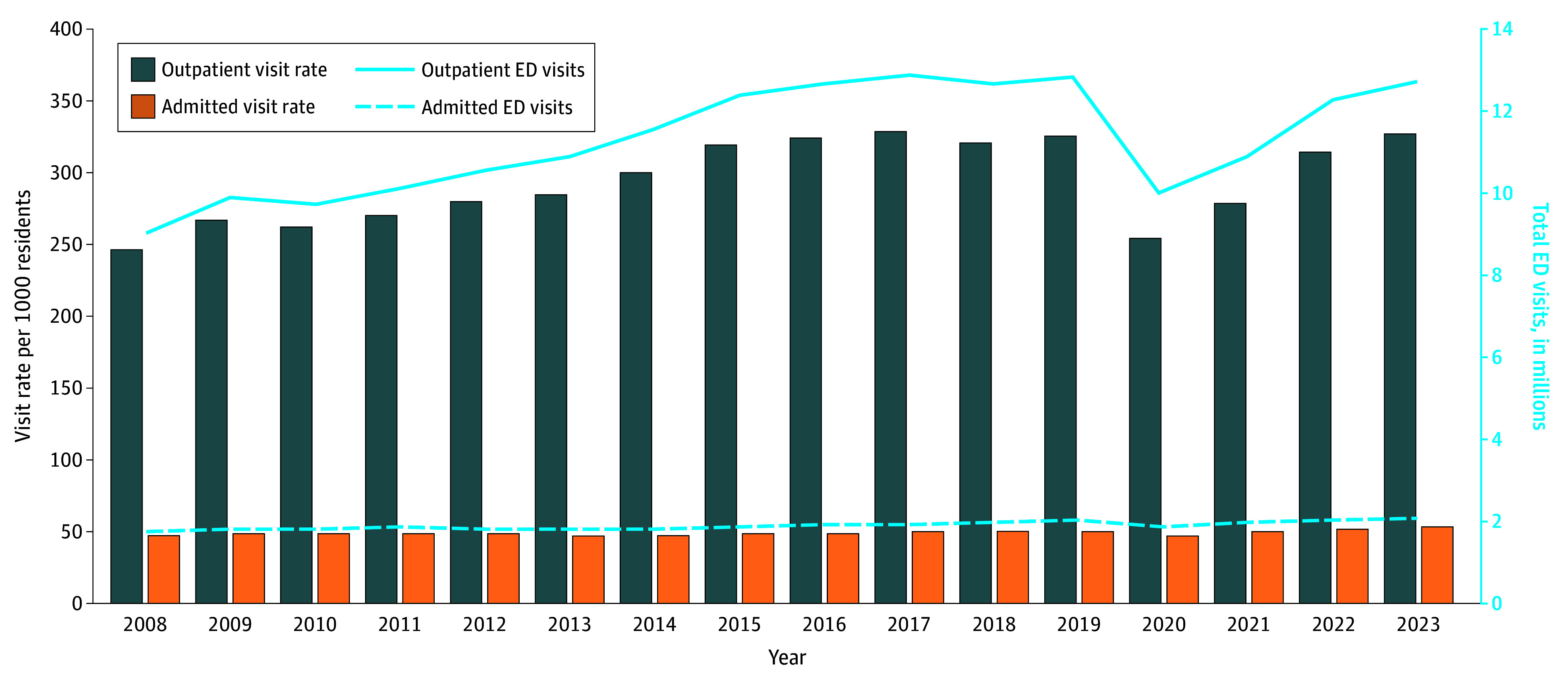
Outpatient Emergency Department (ED) Visit Growth vs Inpatient Trends From 2008 to 2023

Discharge visits per 1000 enrollees increased across all major payers, with the largest relative increases among Medicaid (507.2 to 698.0, a 38% increase) and Medicare (302.4 to 386.1, 28% increase). More modest increases occurred among privately insured (8.0%) and uninsured (6.7%) individuals. In contrast, admitted visit rates decreased dramatically by 45% for uninsured individuals, 16.5% for privately insured individuals, 12.8% for Medicare patients, and 3.3% for Medicaid patients.

## Discussion

Discharged ED visits increased across all payer types, outpacing California’s population growth of 6.4% during the period (from 36.7 to 39.1 million). In contrast, admission rates declined or remained stable, suggesting a rising hospitalization threshold and an expanding role of the ED in outpatient-level care.^1,5^

These patterns are striking, given Medicare’s near-universal coverage. Rising outpatient use among this well-insured population challenges the assumption that coverage alone is associated with reduced ED reliance.^1,2,3^ Similar trends among privately insured and Medicaid patients reinforce that systemic forces beyond payer-specific factors are associated with these trends. This rise occurred during major coverage changes: Medicaid enrollment doubled, and uninsurance fell by more than 50%. Yet per capita ED use among Medicaid enrollees rose by only 38%, suggesting that expanded coverage may have reduced barriers to non-ED care, while uninsured visits declined by 45%, trends that were consistent with coverage expansion. The COVID-19 pandemic also altered ED use, with deferred or shifted care likely contributing to discharge and admission changes.^6^ Beyond coverage, other system-level factors, including regional clinician shortages, rural hospital closures, and higher admission thresholds,^6^ likely shaped these patterns. Shifts from admitted to discharged encounters also carry financial implications, as Medicare and Medicaid remain central to hospital reimbursement and influence administrative decision-making. As the ED increasingly functions as a site for unscheduled or after hours care, policy responses focused solely on coverage may overlook operational and access drivers.^5^ Limitations included a reliance on California data, absence of geographic stratification, and lack of observation capture. Our aggregate categories mask heterogeneity (dual eligibility, Medicare Advantage, and Veterans Affairs [VA] policy changes). Patients with other/unknown insurance represent a diverse group, including limited-benefit, military/VA, or incomplete payer data.

## Notes

Supplement. Data sharing statement
