# Supplementary material for: Outpatient Emergency Department Use Among Publicly Insured Patients
Source: JAMA Health Forum. 2025 Dec 26;6(12):e255916. doi: 10.1001/jamahealthforum.2025.5916 (PMC12743278; doi:10.1001/jamahealthforum.2025.5916)
Supplement: Supplement. — Data sharing statement [file jamahealthforum-e255916-s001.pdf]

Data Sharing Statement

Spencer. Outpatient Emergency Department Use Among Publicly Insured Patients. *JAMA Health Forum*. Published December 26, 2025. doi:10.1001/jamahealthforum.2025.5916

Data
Data available: No
